# Interoperability of CABRI Services and Biochemical Pathways Databases

**DOI:** 10.1002/cfg.376

**Published:** 2004-03

**Authors:** Paolo Romano, Ottavia Aresu, Maria Assunta Manniello, Barbara Parodi

**Affiliations:** IST Istituto Nazionale per la Ricerca sul Cancro c/o Centro Biotecnologie Avanzate Largo Rosanna Benzi 10 Genova I-16132 Italy

## Abstract

Common Access to Biological Resources and Information (CABRI) service is a ‘one-stop-shop’ for materials that are collected by a number of European culture collections that engage themselves in a quality service for the scientific community by adhering to Quality Guidelines for the management of resources and related information. It includes collections' catalogues that can be searched in an SRS implementation. A simple search facility, including a synonym search and a shopping cart, is also available. Within the European Biological Resource Centres Network (EBRCN) project, an extension and improvement of the catalogues' information is under way. This includes adding links to bibliographic databanks and sequence databases. Revision of ‘in-house’ controlled vocabularies used by data annotators is under way, in order to improve the setting up of external links, and new links to biochemical pathways databases are being set up for some of the catalogues.


**Comparative and Functional Genomics**
*Comp Funct Genom* 2004; **5**: 169–172.
Published online in Wiley InterScience (www.interscience.wiley.com). **DOI:** 10.1002/cfg.376

Conference Review

### Interoperability of CABRI services and biochemical pathways databases

Paolo Romano*, Ottavia Aresu, Maria Assunta Manniello and Barbara Parodi
*National Cancer Research Institute, Genova, Italy*

*Correspondence to: Paolo Romano, IST Istituto Nazionale per la Ricerca sul Cancro, c/o Centro Biotecnologie Avanzate, Largo Rosanna Benzi 10, I-16132 Genova, Italy. E-mail: paolo.romano@istge.it*

Received: 7 November 2003
Accepted: 24 November 2003

### Abstract

**Common Access to Biological Resources and Information (CABRI) service is a 'one-stop-shop' for materials that are collected by a number of European culture collections that engage themselves in a quality service for the scientific community by adhering to Quality Guidelines for the management of resources and related information. It includes collections' catalogues that can be searched in an SRS implementation. A simple search facility, including a synonym search and a shopping cart, is also available. Within the European Biological Resource Centres Network (EBRCN) project, an extension and improvement of the catalogues' information is under way. This includes adding links to bibliographic databanks and sequence databases. Revision of 'in-house' controlled vocabularies used by data annotators is under way, in order to improve the setting up of external links, and new links to biochemical pathways databases are being set up for some of the catalogues.**
**Copyright © 2004 John Wiley & Sons, Ltd.**

**Keywords:** **biological material; biological resource centres; biological resource databases; biochemical pathways databases; SRS; data integration**

### Introduction

The definition of Biological Resource Centres (BRCs) by the Organisation for Economic Cooperation and Development (OECD [8]) is:

> Biological Resource Centres are an essential part of the infrastructure underpinning biotechnology. They consist of service providers and repositories of the living cells, genomes of organisms, and information relating to heredity and the functions of biological systems. BRCs contain collections of culturable organisms (e.g. microorganisms, plant, animal and human cells), replicable parts of these (e.g. genomes, plasmids, viruses, cDNAs), viable but not yet culturable organisms cells and tissues, as well as databases containing molecular, physiological and structural information relevant to these collections and related bioinformatics.

The main scope of BRCs is the collection, quality control, characterization, expansion and redistribution of living biological materials and information of controlled quality. Information management is thus an essential part of the service offered by these centres. Integration of information held by BRCs, including catalogues, in the emerging bioinformatics distributed network environment will allow a global viewing of all existing data, and will make the holdings in the collections more effectively available after a search in biological databanks. This integration will also make it easier to retrieve detailed information from molecular biology databanks after a search in the catalogues of the collections. As a major consequence, this will imply that additional certified information will be made available to researchers. Moreover, a growing number of researchers will hopefully refer to BRCs and make a wider use of biological material of certified quality, thus raising the level of today's biological research.

### Common Access to Biological Resources and Information (CABRI)

CABRI [3] is a demonstration project that was initially funded by the European Union (EU) in

Copyright © 2004 John Wiley & Sons, Ltd.

1996–1999. Its main goals are to increase awareness among scientific users of the quality and variety of European culture collections and to facilitate access to information and material.

In order to reach these objectives, the project has implemented a unified access to the culture collection catalogues of participating collections and guaranteed a common level of quality of material and related information. The final achievement of the project has been the development of an on-line 'one-stop-shop' for biological resources where researchers can search, analyse, identify, select and pre-order strains of interest (**http://www.cabri.org/**).

CABRI online services allow the user to check on the availability of a particular item, by interrogating one or more catalogues at the same time, and to pre-order the retrieved biological resources, once located.

The CABRI system is based on the Sequence Retrieval System — SRS [5,13]. Catalogues have been implemented in SRS by first comparing the data structure and contents of collections' internal databases and then defining three distinct datasets for each material.

The minimum dataset (MDS) consists of mandatory information needed to identify a unique item in a catalogue: elements from a collection for which this information is not available cannot be inserted into the catalogue, since they are lacking some essential data.

The recommended dataset (RDS) includes supplementary information that is useful in order to achieve an improved description of the characteristics, functions and properties of the material. Although not mandatory, these data should always be included in the catalogue, when available.

The full dataset (FDS) provides all remaining available information related to the material. Since the original CABRI catalogues were independently built, they do not share a common FDS and each collection can have its own FDS; nevertheless, the corresponding information undergoes a homogenization effort.

Data input procedures define each field of the MDS and RDS by providing a detailed textual description of its contents and by specifying the input process for the corresponding values. This process can foresee the use of a reference list of agreed values or vocabularies, and/or a predefined syntax for insertion of the data (e.g. for bibliography). Special attention is devoted to the information that is important for the implementation of links between catalogues and with external databanks.

### European Biological Resource Centres Network (EBRCN)

With the termination of the EU-funded period, CABRI member collections considered it necessary to maintain the CABRI online service and decided to secure its further existence. Since 2001, CABRI is the core activity of the European Biological Resource Centres Network (EBRCN [4]), which is formed by most of the former CABRI members plus additional collections.

An important goal for EBRCN is to introduce new information technology tools to add value to current catalogue information and enhance accessibility. This will mainly be implemented by linking catalogue data to bioinformatics resources, such as sequence and genetic databases, and to literature databanks. Links to Medline are already in place for some catalogues, while a general procedure to identify and set up links between the EMBL Data Library and the CABRI catalogues has been defined and will start soon.

The catalogue information, which is a basic resource, will also be offered for linking to other non-EBRCN services so as to maximize the use of the data and eventually offer users access to a series of validated information resources. Limited, significant information will therefore be periodically extracted from the catalogues and made available for downloading to interested public services.

### Interoperability with biochemical pathways databases

Links to and from biological pathways databases can actually be useful for BRC users and other researchers. While CABRI does not yet include any such link, plans exist within the EBRCN project for extending cross-references with other external databases as much as possible. It is essential to consider that some information regarding properties of resources which are useful for the study of pathways is already available in CABRI catalogues. As an example, consider that the description of a

Copyright © 2004 John Wiley & Sons, Ltd. 

number of animal cell lines, bacteria and fungi in CABRI catalogues already refers to the use of the items for studying enzymatic pathways. This information is included in the fields *Enzyme production* and *Metabolite production* for microorganisms and *Further information* and *Properties* for other materials. Table 1 reports some examples, including enzymes, other proteins, chemical compounds and genes.

It is therefore possible to establish an effective link from such records in CABRI catalogues to corresponding records in many external databases that are of interest for the analysis of biochemical pathways. Attention has therefore been given to the Enzyme [1], BRENDA [12], TransFac [7] and TransPath [11] databases. The two latter systems already include links to cell lines that are described in the Cell Line Data Base (CLDB) [2,10]. Links to the CABRI catalogues of human and animal cell lines are also foreseen.

Ongoing work is aimed at identifying all biological agents listed in CABRI catalogues, checking terms in the descriptions, identifying related unique identifiers in the above-listed databases and adding them to the respective record in CABRI catalogues.

The result of this effort will allow for a quicker and more effective link between CABRI catalogues and biochemical pathways catalogues, especially when these are available in the same SRS environment.

Within the CABRI catalogues of human and animal cell lines, the properties of cultures are defined on the basis of controlled vocabularies. A revision of this thesaurus is also being performed in order to offer a better description of cell lines' usefulness in studies on biochemical pathways.

Finally, a further characterization of a panel of cell lines from the Italian collection of the Cell Bank of the National Cancer Research Institute of Genova (Interlab Cell Line Collection — ICLC [6,9]) is foreseen. This characterization would be aimed at describing metabolic changes in growing cell cultures by using an ISFET biosensor (which is able to measure medium acidification in minimal volumes) to monitor the metabolic activity of small numbers of cells, in response to specific stimuli.

### Conclusions

Biological resources are essential tools for biomedical research today. In order to improve access to microorganisms and cells of certified quality, the CABRI services were set up as a one-stop-shop for European quality materials. Integration of this information into the bioinformatics network environment is under way and more and more links to and from some of the main Bioinformatics databases are being added to CABRI catalogues. Links to some biochemical pathways databases are being added. A revision of controlled vocabularies describing properties of cell lines has been performed in order to offer a better description of their usefulness in studies on biochemical pathways. An extended characterization of biochemical changes occurring in cell lines in culture is foreseen for a panel of cell lines. These improvements promise to

**Table 1.** Example list of biological items included in CABRI catalogues which are of interest for studies on biochemical paths

| Catalogues | Field | Contents | Example |
|---|---|---|---|
| Cell lines | Properties | Products | Products: acetylcholinesterase, choline acetylase, tyrosine hydroxylase |
| Cell lines | Comments | Products | Cells have characteristics of mature neurons and secrete the enzymes for the synthesis of the neurotransmitters, choline acetylase and tyrosine hydroxylase |
| Bacteria | Metabolite_production | Chemical compounds | 3′-Phosphoadenocyne-5′-phosphosulphate; adenosine 5′-phosphosulphate |
| Bacteria | Enzyme_production | Enzymes | 20-$\beta$-Steroid dehydrogenase; 20-$\beta$-ketoreductase |
| Bacteria | Applications | Products | Production of 2,3-butanediol; production of glycerol; production of $\alpha$-acetolactate decarboxylase; production of $\alpha$-amylase (thermostable); production of $\beta$-lactamase |

Copyright © 2004 John Wiley & Sons, Ltd. 

offer a better integration of CABRI catalogues with biochemical pathways databases.

### Acknowledgements

This work was partially supported by the European Union through the European Biological Resource Centres Network — EBRCN project (Contract Nos QLRI-CT-2000-00221 and QLRT-2001-02806). We also wish to thank all participants in the European Science Foundation Workshop on 'Data integration in functional genomics and proteomics: application to biological pathways', held in Geneva on 22–24 September 2003, for the contribution of ideas given during the workshop.

### References

1. Bairoch A. 1993. The ENZYME data bank. *Nucleic Acids Res* **21**(13): 3155–3156.
2. Cell Line Data Base (CLDB): **http://www.biotech.ist.unige.it/interlab/cldb.html**
3. Common Access to Biological Resources and Information (CABRI): **http://www.cabri.org/**
4. European Biological Resource Centres Network (EBCRN): **http://www.ebrcn.org/**
5. Etzold T, Ulyanov A, Argos P. 1996. SRS: information retrieval system for molecular biology databanks. *Methods Enzymol* **266**: 114–128.
6. Interlab Cell Line Collection (ICLC): **http://www.iclc.it/**
7. Matys V, Fricke E, Geffers R, *et al.* 2003. TRANSFAC: transcriptional regulation, from patterns to profiles. *Nucleic Acids Res* **31**(1): 374–378.
8. OECD. 2001. Biological Resource Centres; Underpinning the Future of Life Sciences and Biotechnology: Reports of the Organisation for Economic Cooperation and Development: **http://www.oecd.org/**
9. Parodi B, Aresu O, Visconti P, *et al.* 2000. The Interlab Cell Line Collection (ICLC). In Cell Banks: a service to animal cell technology. Stacey G, Doyle A (eds). In *Encyclopedia of Cell Technology*, Spier R (ed.). Wiley: New York; 313–314.
10. Romano P, Iannotta B, Rondanina G, Ruzzon T. 1992. The Interlab Project Network for Biomedical Research. *Bioinformatics* **1**(3): 4–11.
11. Schacherer F, Choi C, Götze U, *et al.* 2001. The TRANSPATH signal transduction database: a knowledge base on signal transduction networks. *Bioinformatics* **17**: 1053–1057.
12. Schomburg I, Chang A, Schomburg D. 2002. BRENDA, enzyme data and metabolic information. *Nucleic Acids Res* **30**(1): 47–49.
13. Zdobnov E, Lopez R, Apweiler R, Etzold T. 2002. The EBI SRS server — new features. *Bioinformatics* **18**(8): 1149–1150.

Copyright © 2004 John Wiley & Sons, Ltd. 
